# Insights into Circular Horticulture: Knowledge Diffusion, Resource Circulation, One Health Approach, and Greenhouse Technologies

**DOI:** 10.3390/ijerph191912053

**Published:** 2022-09-23

**Authors:** Diego Alejandro Salinas-Velandia, Felipe Romero-Perdomo, Stephanie Numa-Vergel, Edwin Villagrán, Pilar Donado-Godoy, Julio Ricardo Galindo-Pacheco

**Affiliations:** 1 Corporación Colombiana de Investigación Agropecuaria–AGROSAVIA, C.I. Tibaitatá, Mosquera 250040, Cundinamarca, Colombia; 2 Global Health Research Unit for the Genomic Surveillance of Antimicrobial Resistance (GHRU–Colombia), CI Tibaitatá, Corporación Colombiana de Investigación Agropecuaria (AGROSAVIA), Mosquera 250040, Cundinamarca, Colombia

**Keywords:** circular economy, sustainability, One Health, food systems, bibliometrics, biomass, greenhouse structure

## Abstract

The integration of the circular economy in agriculture has promoted sustainable innovation in food production systems such as horticulture. The present paper illustrates how horticulture is transitioning to the circular economy. This research field’s performance approaches and trends were assessed through a bibliometric and text-mining analysis of the literature. Our findings revealed that circular horticulture is a recent research field that is constantly growing. Its approach has been neither systemic nor integrative but fragmented. Bioeconomy, urban agriculture, recycled nutrients, biochar, fertigation, and desalination have been positioned as research hotspots. Vegetables and fruits are the most studied crops. Resource circulation has focused primarily on biowaste recovery to provide benefits such as biofertilizers and linear-substrate substitutes, and on water reuse for the establishment of hydroponic systems. The One Health approach is scarcely explored and, therefore, weakly articulated, wherein the absence of assessment methodologies encompassing the health of ecosystems, animals, and people is a notable limitation. Science-policy interfaces between One Health and food systems need to be improved. Lastly, greenhouse technologies are aligned with bioenergy, sustainable materials, and sensing technologies. Challenges and directions for future research have been raised to promote the redesign of horticultural production systems, integrating long-term circularity.

## 1. Introduction

Food waste and loss are global issues affecting agriculture systems’ sustainability. It is estimated that approximately 35% of the food produced for human consumption is lost or wasted [1]. Food wastes generate approximately 8% of global greenhouse gases, occupy 23% of all croplands, and consume approximately 25% of all water used by agriculture annually [2]. Consequently, disturbances of fundamental earth system processes and the transgression of planetary boundaries such as overexploitation of natural resources, soil–air–water pollution, altered biogeochemical cycles, changes in land use, and loss of biosphere integrity have increased [3]. Prospects point to these effects becoming more pronounced as agricultural production must be increased to ensure food security for a growing population [4]. Key strategies will thus need to be deployed on a large scale to promote resource sufficiency by addressing agricultural waste and food production in a less-polluting way [5].

The circular economy proposes a paradigm shift that allows slowing down the natural deterioration of the planet through regenerative systems of material, water, and energy cycles [6]. The circular economy is a model that seeks to close cycles of the traditional linear economy pattern that follows the take–make–dispose scheme [7]. Relevant potential thus exists for the circular economy to be applied in the agri-food sector. It implies systemic thinking to reduce the waste generated, valorize waste, reuse food, recycle nutrients and water, and establish more sustainable diets [8]. The integration of the circular economy in agriculture has led to the emergence of circular agriculture as a new field of research. Today, circular agriculture is being implemented worldwide, from small agricultural fields to large countries, mainly in Europe [9]. Although the challenge of consolidating circular agriculture continues, the generation of new knowledge that standardizes indicators, processes, and products has recently been reported [10].

Horticulture, seen as a subfield of agriculture, has shown progress in adopting circular principles. The use of greenhouse structures in horticulture has been relevant for circularity due to their high potential for recycling water and nutrients, their higher production capacity compared to open field-based agriculture per hectare, and their high productivity with reduced use of water and agrochemicals per production unit [11]. Research linking the circular economy and horticulture has focused on resource circulation and greenhouse technologies for intensive agricultural production from protected crops [12]. Examples are the use of circular organic fertilization, the implementation of by-products in crop fertigation, the application of wood fiber as an organic substrate to replace peat, and the launch of public policies for sustainability and water security [13]. A minority of growers even use the recirculation of nutrients and water in the Almeria region, especially to cultivate tomatoes and lettuce [14].

The transition to a circular model has intensified several health risks of reusing by-products, components, and materials, where the horticultural sector is no exception [15]. The recovery of animal waste is the main critical point [16,17]. Research in this context has been aligned with techniques to inactivate pathogens, eliminate veterinary antibiotics, reduce potentially harmful agrochemicals in food, and investigate the microbiome and resistome transmission pathways from manure to soil and crops [18,19]. However, the health effects are only partially understood, and much more comprehensive evidence to better inform the policy debate is lacking [20]. In response, the ‘One Health’ concept drives the assessment of the health impacts of the circular economy. This concept promotes balanced well-being between humans, animals, and the environment, providing a global strategy that highlights the need for holistic and transdisciplinary approaches to improve the connection of all components of an ecosystem [21]. Horticulture presents an intrinsic closeness to this tripartite interaction, playing an essential role as food represents the link between soil, plants, and animals for human health [19]; therefore, circular horticulture, especially for the valorization of animal biomass, can be strongly influenced by the One Health concept. The need for circular practices based on One Health is expected to increase in the future [22].

Circular horticulture is increasingly recognized in sustainable agriculture transitions. However, the lack of systemic efforts to map the existing scientific literature on circular horticulture led us to ask what the research advances on the circular transition of horticulture are. Consolidating its state-of-the-art and identifying its actors is key to guiding future initiatives, projects, and approaches [23]. Bibliometrics allows the unraveling of evolutionary nuances of a specific field while shedding light on emerging areas in that field [24]. This is complemented by text mining that identifies patterns or correlations between terms, exploring the dynamics of the information in the publications [25]. The present work aimed to explore trends in circular horticulture research to provide an overview of the publication landscape and knowledge structure from a bibliometric and text-mining perspective. Four research questions were defined, motivated by the dissemination of knowledge and topics, circulation of resources, the One Health approach, and greenhouse technologies. The research questions were the following:

(i) How has the dissemination of circular horticulture knowledge evolved worldwide, including countries, affiliations, and authors?
(ii) What are the salient research topics of circular horticulture?
(iii) What is the trend of crops, resource circulation strategies, implications of One Health, and greenhouse technologies in circular horticulture research?
(iv) What are the challenges and directions for future research on horticulture toward the circular economy transition?

The presented work represents an interdisciplinary analysis that helps to design more specific interventions to close the circular gap in horticulture. Likewise, it serves as a reference document for various actors in environmental research and public health, sustainable horticultural production, and government decision makers.

## 2. Materials and Methods

The methodological structure of this study was carried out in three steps. The first step consolidates the literature on circular horticulture by defining the search equation, selecting the database, and filtering publications. The second step represented the bibliometric analysis with the numerous parameters used in the publications. The third step was text mining applied to relevant topics of circular horticulture reported in the literature. 

### 2.1. Data Sampling, Collection, and Cleaning

The search equation was composed of two parentheses with the main keywords of the two associated topics together and four Boolean operators (“ “, OR, AND, *). To create an overview of the impact of the circular economy on agriculture and horticulture, we used Equations (1) and (2), respectively.

((“food system*”) OR (agr*)) AND ((“circular *economy”) OR (“circular agr*”))(1)

(horti*) AND ((“circular *economy”) OR (“circular horticult*”))(2)

The database was selected by comparing Web of Science and Scopus using the total number of publications as a parameter. These two databases mostly cover a wide range of research fields and are therefore widely used to perform multidisciplinary analyses [26]. For the first search equation, 1488 and 1731 publications from Web of Science and Scopus were examined, while for the second, 59 and 73 publications were examined, respectively. Hence, Scopus contained a larger number of publications and was selected. As we sought to consolidate the research reported to date bibliometrically, we did not define a range of years for the search. The search period was limited solely by the coverage of the databases. The search was carried out in a single day (2 March 2022), with “subject” as the scope, which included the title, abstract, and keywords. During the search in the databases, research and review articles were selected, all available in English.

We used three types of sequential filters to select the final set of circular horticulture publications, as follows: (i) exclude duplicates, (ii) exclude publications with an abstract that were unrelated to the topic, and (iii) exclude publications that were cost reasons could not be downloaded. Therefore, the 73 publications were reduced to 67 (Table S1), exported as comma-separated value (csv) files and imported into Microsoft Excel 2016.

### 2.2. Bibliometric Analysis

Bibliometric parameters associated with scientific productivity were used in this study. The parameters at the general level of the research field were the number of types of documents (research articles and review articles) and the number of publications per year. The country parameters were the number of publications, citations, and keyword frequency. The scientific production was measured by the frequency of the countries associated with the authors’ affiliations in the publications. The parameters by institution and authors were the number of publications per institution, the number of publications per author, the annual number of publications per author, and Lotka’s law. Keyword dynamics were shown by keyword frequency in all publications and keyword co-occurrence. All these parameters were provided by Bibliometrix software (version 4.0.0, R Core Team).

Particularly, Lotka’s law denotes the number of authors by the number of publications to understand productivity patterns. This indicates that the relative frequency of authors (y) with publications (x) can be described by the following Equation (3):

y = c/x^n^ (3)

where c is constant, and n approximately equals 2 [27].

The keyword co-occurrence map shows the connection between keywords. Co-occurrence is measured when two keywords appear in the same document. The most reported keywords were extracted from the publications’ title and abstract using the complete count method and were selected for a minimum of five occurrences. Subsequently, Bibliometrix generated the map of co-occurrence with grouped keywords and the figure associated with the frequency of keywords in all publications. The other figures related to bibliometric parameters were performed with Prism 8 software (Graphpad, San Diego, CA, USA).

### 2.3. Text-Mining Analysis

**Figure 1:**
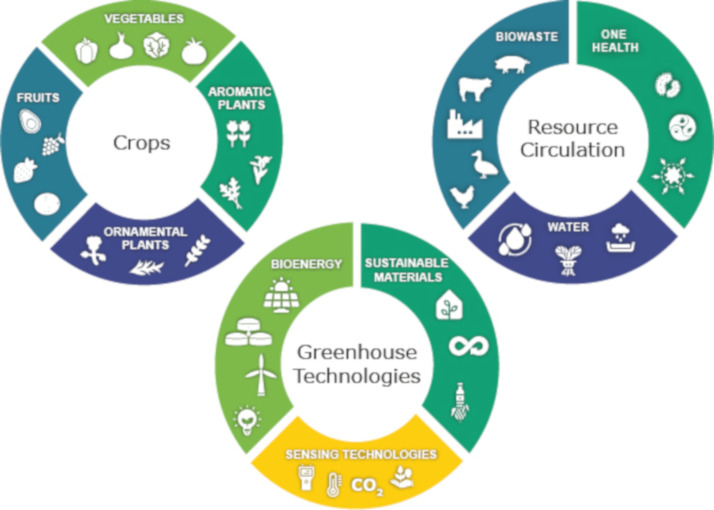
Text-mining analysis framework on the trend of relevant topics in circular horticulture—own elaboration.

The topics for the text-mining analysis were associated with horticultural crops, resource circulation, and greenhouse technologies (Figure 1). The crops were searched through the categories of vegetables, fruits, ornamental plants, and aromatic plants. The resources were addressed from the biowaste reuse, water management, and One Health implications. Greenhouse technologies were focused on sensing technologies, sustainable materials, and bioenergy.

The normalized relative frequency of the specific topics per year was calculated using an in-house Python script (available at https://github.com/LeonardoMorenoG/textMiningCH.git; accessed on 9 May 2022). Publications in PDF format were first parsed to text using the package pdfminer (https://github.com/euske/pdfminer.git, accessed on 10 July 2022). Any punctuation signs were removed, and all uppercase letters were converted to lowercase before counting the frequency of each keyword related to each specific topic (Table S2). The relative frequency, *R*, of the *i*th specific topic at a year, *t*, was then calculated as:

(4) Ri,t=∑j=1qinjqi* Nt

where *q_i_* is the number of keywords associated with the specific topic, *i*, *n_j_* is the number of occurrences of its *j*th keyword, and *N_t_* is the total number of words in all publications at year *t*, as reported by Dayeen et al. [28]. Finally, a relative frequency heatmap was generated using seaborn and customized using matplotlib.

## 3. Results

### 3.1. Interest in Research on Circular Agriculture and Horticulture

**Figure 2:**
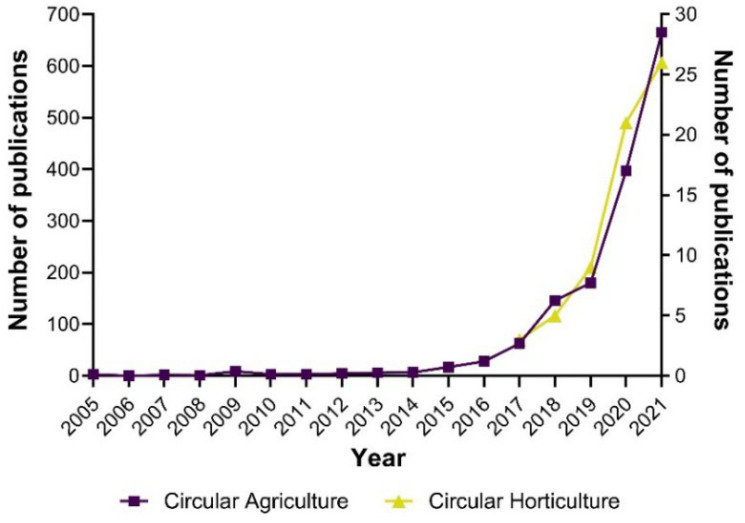
Annual publication trend on circular agriculture and circular horticulture from Scopus database. The number of publications per year is the sum of original articles and reviews. The left Y axis of the figure corresponds to circular agriculture, while the right Y axis corresponds to circular horticulture—own elaboration.

The computational analyzes of the literature, such as bibliometrics and text mining, have contributed substantially in the last decade to the profound review of scientific knowledge. To understand the impact of the circular economy on horticulture, we compiled insights on knowledge dissemination and research progress from a bibliometric perspective. Firstly, we explored the interest in research on applying the circular economy to agriculture and specifically to the horticultural sector through the number of publications per year. In this sense, we have addressed the first research question on knowledge worldwide dissemination. We found that circular agriculture research led to 1731 publications, wherein 77% were original and 33% were review articles (Figure 2). Circular horticulture research has led to 73 publications, with 80% and 20% original articles and reviews, respectively. This comparison indicates that circular horticulture research represents approximately 4% of circular agriculture. Moreover, circular agriculture presented its first report 17 years ago, while circular horticulture presented 5 years ago. Despite this notable difference, circular agriculture publications have grown sharply since 2017. Interestingly, the largest increase for both research topics was seen during the period 2020–2021.

### 3.2. Most Outstanding Countries

**Figure 3:**
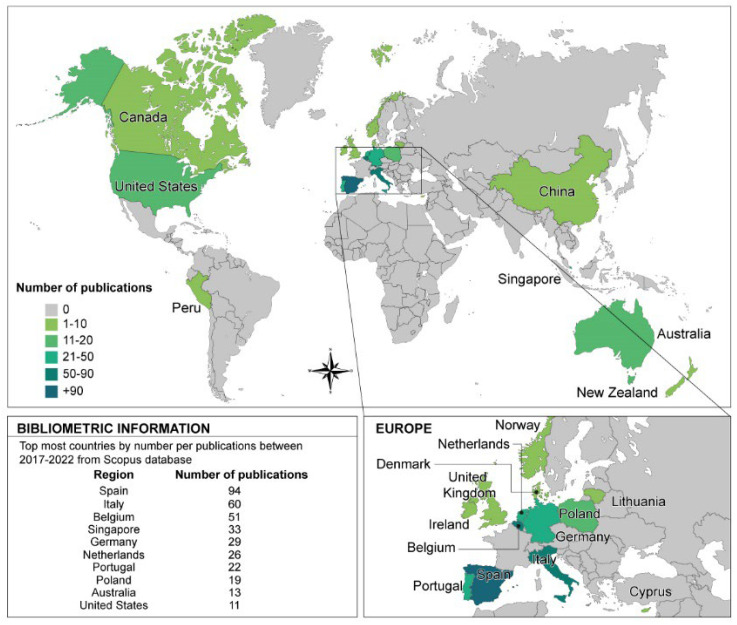
Geographical dynamics of most productive countries in the research of circular horticulture—own elaboration.

Next, we examined the scientific production of the most relevant countries in circular horticulture to understand the research dynamics in a geographical context and thusly continue to answer our first research question. The scientific production was measured by the frequency of the countries associated with the authors’ affiliations in the publications. Thirty-nine countries worldwide are researching circular horticulture (Figure 3). Notably, Spain is the most productive country. Italy and Belgium are in second and third place, respectively, with a frequency of one-third and one-half compared to Spain. Singapore, Germany, the Netherlands, Portugal, Poland, Australia, and the USA complete the top 10 in their respective order. Therefore, the continent that unquestionably leads is Europe. The remaining countries, from 11th to 20th place, are Denmark, Lithuania, Norway, Canada, China, the UK, Cyprus, Ireland, New Zealand, and Peru.

**Figure 4:**
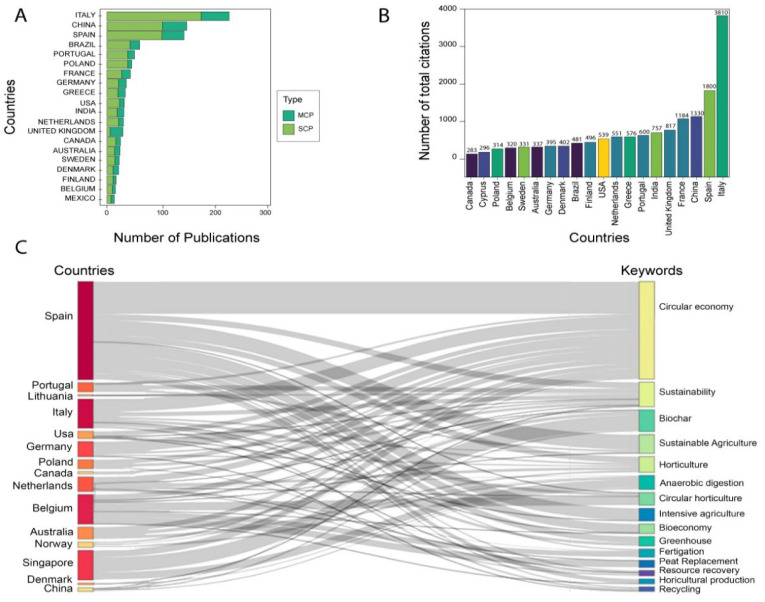
Geographical dynamics of research in circular horticulture. Cooperation between countries (**A**), most cited countries (**B**), and most reported keywords by country (**C**)—own elaboration.

Country cooperation was assessed by measuring whether a single country or multiple countries appeared in a publication. We noted that Italy ranks first, with the largest number of both single-country publications and multiple-country publications (Figure 4A). China and Spain rank second and third, respectively, in both types of cooperation. Interestingly, single-country publications dominate the entire ranking. The total number of citations in the countries was also analyzed. Italy is the most-cited country in its publications, with 3810 citations (Figure 4B). With less than half of the citations are the other countries, wherein Spain, China, and France complete the ranking of countries that exceed 1000 citations. The UK is fifth with 817 citations and the USA is tenth with 539. Additionally, we described the topics that the countries are researching through the association with the keywords reported in a Sankey diagram (Figure 4C). Spain is focusing its research on the circular economy, sustainable and intensive agriculture, horticulture, and bioeconomy. Italy and Germany also contribute markedly to reporting on the circular economy. Singapore is the country that has the most reports regarding biochar and anaerobic digestion, while Belgium has the most reports regarding circular horticulture, fertigation, peat replacement, and greenhouses. Finally, resource recovery and recycling are promoted in publications mainly by Spain, Italy, and Germany.

### 3.3. Most Prolific Institutions and Authors

**Figure 5:**
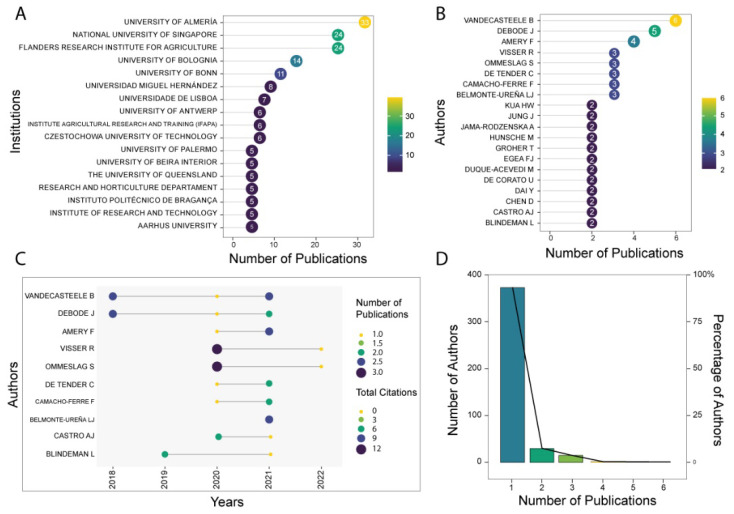
Publications productivity of institutions and authors on circular horticulture. Top 10 institutions with the highest number of publications (**A**), Top 10 authors with the highest number of publications (**B**), production over time of the authors (**C**), and use of the Lotka’s Law (**D**)—own elaboration.

We also analyze the scientific production of the most relevant institutions and authors to know their cooperation interactions and provide the main references of circular horticulture. The top 10 affiliations present a range of publications between 33 and 6 (Figure 5A). The most productive affiliation in circular horticulture is the University of Almería. Then there are the National University of Singapore, Flanders Research Institute for Agriculture, Fisheries and Food, the University of Bologna, and the University of Bonn, with over ten publications.

In regard to authors, we observed slight differences in the top 10 (Figure 5B). Bart Vandecasteele is the leader in publications. Interestingly, the second and third authors are Jane Debode and Fien Amery. There are then five authors tied with three publications: Luis Belmonte-Ureña, Francisco Camacho-Ferre, Sarah Ommeslag, Caroline De Tende, and Rian Visser. Over time, the top authors showed that Bart Vandecasteele and Jane Debode have the most constant productivity in the five years of circular horticulture (Figure 5C). Luis Belmonte-Ureña and Francisco Camacho-Ferre stand out for their productivity and citations in the last two years. Finally, we analyze the authors using one of the main pillars of bibliometric analysis: Lotka’s law. This law indicates the distribution of authors during a specific period or within certain subject areas. One interpretation that can be deduced, for instance, is that many authors publish solely one study, while a small group of prolific authors contribute many publications. We found a dramatic decrease in the number of authors as the number of publications increased (Figure 5D). The largest concentration of authors (379) reported one publication, representing 92%. Two publications have been written by 24 authors (5%), while five authors have three publications. From four to six publications, one author was found. Based on the findings found in the institutions and authors, we finished answering the first research question.

### 3.4. Research Hotspots: Keyword Frequency and Cooccurrence

**Figure 6:**
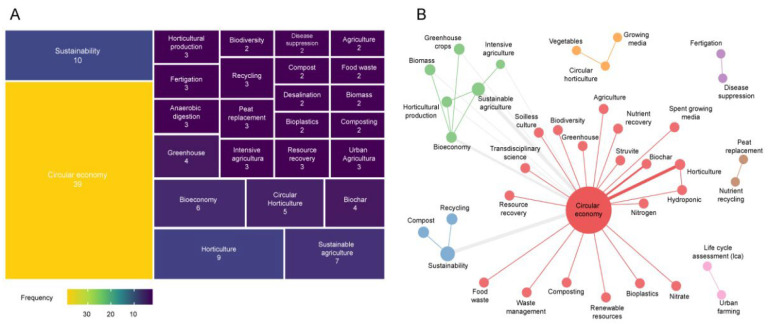
Use of keywords in research on circular horticulture. Frequency and percentage of the top 25 keywords (**A**) and co-occurrence map (**B**)—own elaboration.

Because keywords denote salient research topics, we next explored the trend of keyword usage to determine what they reveal, thus answering the second research question. The most-used keyword in circular agriculture research is circular economy, with 31%, as expected (Figure 6A). Sustainability ranks second, less than a third (8%) as often as circular economy. This is then followed by sustainable agriculture, bioeconomy, circular horticulture, biochar, and greenhouse, between 6% and 3%. The remaining keywords (i.e., composting, bioplastics, fertigation, desalination, etc.) in the top 25 have 2%.

We constructed a co-occurrence map to study how keywords interact. The occurrence was the parameter that allowed description of the interaction. Two words are defined as co-occurring if they appear in the same document. The size of the node represents the frequency of the keyword and the thickness of the link the co-occurrence, which generated seven clusters, distinguished by colors (Figure 6B). The keywords that dominated by cluster were circular economy (red), sustainability (blue), sustainable agriculture (green), nutritional recycling (brown), and circular horticulture (orange); fertigation and disease suppression are tied (purple), as are life cycle assessment and urban farming (pink). Likewise, the red cluster displays the largest size in the number of keywords, followed by the green one. Strong links were noted between circular economy and sustainable agriculture, circular economy and horticulture, circular economy and sustainability, circular economy and bioeconomy, and circular economy and biochar.

Additionally, we analyzed the ranking of the 10 most influenced publications, based on their citations (Table S3). The research questions addressed in these publications revealed that barriers in farmers’ perceptions need to be identified, opportunities in related industries improved, and existing circular farming practices transferred.

### 3.5. Text-Mining Findings: Discovering Major Research Topics and Trends

**Figure 7:**
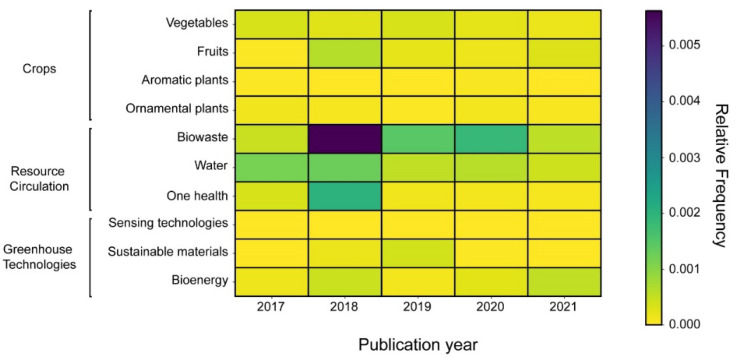
Trends in circular horticulture research topics—own elaboration.

To scrutinize the horticultural literature in the circular economy and address the third research question, we conducted text-mining analyses in the set publications, focussing on three aspects: crops studied, resource circulation strategies used, and implemented greenhouse technologies. The most studied crops were vegetables and fruits (Figure 7). Aromatic and ornamental plants have had a slight prominence. Nevertheless, we did not observe a pronounced growth trend in any crop. Research efforts on resource circulation have focused primarily on biowaste. The most significant relative frequency was obtained with biowaste in 2018, which decreased slightly until 2020. Water circulation is the second-most discussed topic, being the only one that has appeared since 2017. One Health approach displayed a notable prominence in 2018, coinciding with biowaste, but their trend was not constant in the following years. Greenhouse technology was the least prominent focus. Its trend was similar to crops. Bioenergy gave increases in 2018 and 2021, while sustainable materials increased in 2019. In contrast, the inclusion of sensing technology was scarce.

## 4. Discussion

### 4.1. Knowledge Dissemination and Research Progress

A redesign of agricultural production systems based on the circular economy is a promising solution to sustainably transform the food system [29]. In the present study, we consolidated the first literature mapping of reported research linking the circular economy and horticulture. 

Our findings showed that circular horticulture is a recent research field, and its number of publications has grown continuously, yet it still represents a small niche of circular agriculture. Europe markedly leads the dissemination of circular horticulture knowledge in terms of the most prominent countries, institutions, and authors. These results agree with a recent bibliometric mapping, where Italy, Spain, and China stand out in scientific production on circular agriculture, while the United States, China, and Germany are pioneers in crop–livestock systems research, with a strong circular influence [10,30]. Moreover, the geographical centralization of the circular economy worldwide has been found with certainty in Europe in most economic sectors, such as agriculture and horticulture. Government actions of the European Union have placed the circular economy as a pillar of the European Green Deal for its development policy, international cooperation, and research leverage [31].

We noted that vegetables and fruits are the most used crops to investigate circular innovations in horticulture. Lettuce, potato, carrot, onion, tomato, and pak choi are the most reported vegetables, while several studies have used fruits such as apple, avocado, pineapple, papaya, and strawberry. Conversely, ornamental and aromatic plants have been slightly prominent, with reports of *Diplotaxis tenuifolia* and *Valerianella locusta*. Vegetables and fruits play a key role in circular horticulture. From a practical perspective, these account for nearly 90% of global horticulture production, and their consumption can increase substantially by being included in healthy diets, which has shown that their production slightly affects the environment compared to ultra-processed foods [32]. Moreover, the United Nations’ Food and Agriculture Organization (FAO) estimated that higher than 60% of fruit and vegetable waste is reusable, suggesting that the circular management of vegetables and fruits requires intelligent and advanced management for the sustainable reuse of waste that covers its entire life cycle [33].

We found that resource circulation strategies have mainly been applied to biowaste for biofertilizers in nutrient recycling. Its main objective is the reduction of mineral and fossil fertilizers. Biowastes based on alfalfa, lupine meal, castor cake, tomato, tea, fish, algae, cow horn shavings, and cattle and chicken manure are raw materials that demonstrate their biofertilizer potential for both agricultural production and the maintenance of soil health [34,35,36]. Various works have shown that the enrichment of biowaste with microbial biostimulants, biological controllers, and plant extracts is a promising practice to stabilize certain conditions of the biowaste as well as to improve seedling growth further, mitigate abiotic stress, and control pests [37,38,39]. We identified that studies focused on biowaste management are associated with its conditioning, characterization, and application effect in crop production. These methodological approaches are necessary to consolidate biowaste’s nutritional categorization and technical standardization. In spite of this, current valorization techniques must be simplified and improved to massively increase efficiency [40]. However, the economic value, technology transfer potential to other crops, and environmental conditions cannot be ignored [11].

We noted how biowaste has also been investigated to replace the substrates conventionally used under the linear economy model. This is very important, considering that the manufacture and accumulation of conventional single-use substrates carries a sizeable environmental burden, such as greenhouse gas emissions, soil acidification processes, and the depletion of non-renewable resources [41]. Consequently, the reuse of linear substrate or the biowaste that replaces it should be strongly encouraged, together with the reduction of its use quantity [42]. We observed that the biowastes assessed that have led to promising findings are mushrooms, grape pomace, miscanthus, reeds, *Eucalyptus globulus* bark, coconut, wood, flax, and biochar. Their use through composting, vermicomposting, pyrolysis, and anaerobic digestion processes have proven to be a sustainable substitute for peat for horticulture [12,43,44]. It is essential to monitor and record the physical and chemical quality of the substrate to ensure high efficiency in water and fertilizers, which will allow maintaining adequate crop yields so as not to affect food security [45].

Our data suggest that resource circulation strategies have influenced water management to a lesser extent than biowaste. Closed-loop micro-irrigation practices with sensing technologies have been implemented in greenhouses [46]. Research has been advanced in hydroponic systems that improve water management by applying low-cost automated systems based on Raspberry Pi and Arduino to cyclically manage water replenishment [47]. Wastewater recycling as a fertigation method continues to receive attention, but it still represents a bottleneck in standardizing processes with suitable conditions for reuse [48]. Some authors have proposed the cradle-to-cradle system to recycle phosphorus as a heavy metal-free struvite from wastewater to fertilize a lettuce crop [49].

We also found that the coverage of the One Health implications of circular horticulture has been relatively limited. Its approach has been associated with the recovery of biowaste, mainly animal manure [50]. The actions promoted by One Health include improvements in antimicrobial use regulation and policy, infection surveillance and control, and animal husbandry and sanitation [51]. One Health has also fostered the supply of probiotics and postbiotics to manage viral diseases in animals and humans and increased biodiversity by producing of locally adapted crops and livestock breeds [52]. From a research perspective, the integration of quantitative indicators of human health and animal welfare into life-cycle sustainability assessments has been reported. Human health considerations have been dimensioned using epidemiological dietary risk data, expressed as disability-adjusted life years related to the top three diet-related diseases in the study area [53]. For animal health, indicators such as years of animal-life years suffered and loss of animal lives have been proposed [54].

Greenhouse technologies are a fundamental aspect that dramatically influences the efficient and safe production of horticultural crops in a protected environment linear processes, however, have predominated in their operation. We noted that the circular economy is primarily integrated bioenergy sources, followed by sustainable materials and sensitivity technologies in greenhouse structures for horticultural production.

Circular research on energy resources has shown progress in substituting fossil fuels for bioenergy sources to improve the microclimatic management of greenhouses and thus reduce greenhouse gases [55]. This need has drawn attention to the identification that the processes that maintain optimal conditions for plant growth, such as cooling, heating, humidifying, and adding light, emit 96% of the greenhouse gases produced in protected horticulture models [56]. The bioenergy sources implemented are solar, biomass, geothermal, and wind [57]. For example, switching from diesel-based to biomass-based systems for heating greenhouses has substantially reduced heating costs [58]. Biomass sources such as firewood, wood chips, wood pellets, paper pellets, grains (corn, rye), and passive solar techniques are recommended [4].

The most prominent goal of using sustainable materials in greenhouses is to reduce the so-called plastic footprint by developing and implementing bioplastics, long-lasting recycled plastic films, biodegradable plastic films, and compostable plastic films [59]. A similar trend occurs with other plastic materials used in crop management, such as clips, rings, trellis ropes, and mulch plastics [11]. Sensitivity technologies in greenhouses adapted to circularity principles are related to water recycling systems and crop irrigation and fertigation control systems [60]. All these innovations in greenhouse technologies have unfortunately been applied separately and do not form a closed-loop system. Based on this purpose, the GreenFarm model has been reported, which combines greenhouse soilless cultivation, efficient energy conversion technology connected to the greenhouse, and closely cultivated biomass crops [58]. Two benefits of the GreenFarm model are reduced greenhouse heating costs and soil fertility restoration. A third benefit, the most prominent, is using the CO_2_ generated through the biomass-energy conversion process to fertilize crops, which shortens the growth cycle of plants and improves their yield [61,62].

### 4.2. Challenges and Directions for Future Research

The current literature has shown that the circular economy has not been addressed as a system in the entire production cycle of horticulture. Its approach has been fragmented based on evaluating the potential for the reuse of resources. While these assessments are necessary and represent the first steps, circular horticulture requires a systemic transformation involving several challenges that guide the direction of future research, as expressed by our fourth research question. We outline the following recommendations.

Redesigning current horticulture production systems requires holistic approaches that allow the development of both circular flows of resources and circularity indicators. Circular practices do not guarantee sustainability; therefore, their scope and effects must be measured through indicators. Implementing the life cycle assessment with material circularity indicators of the productive system is suggested as a solution strategy. This strategy has recently been conceptualized in the literature and is crucial in improving circular decision making [63]. Likewise, the redesign process requires more extensive automation capabilities to support intelligent decision making based on data [64].

The recovery of biowaste is the hallmark of the circular economy, and there is still a long way to go for horticulture. Challenges have been raised in frequently addressed issues, such as the standardization of processes for the proper conditioning, use, and mixing of biowaste [57]. Nonetheless, some aspects that require research are collecting, transporting, and storing biowaste [65].

The circular approach shows many ecological, social, and economic interdependencies between a system’s actors, drivers, and outcomes. Farmers and consumers are prominent actors who represent a social challenge to reframe that biowaste is a valuable resource [66]. Consequently, we must educate regarding the different productive uses of biowaste and its environmental and economic benefits [67].

The circular transformation of agricultural production systems and the One Health concept should not be seen as isolated issues [19]. We highlight four challenges: (i) its weak research; (ii) the transmission of pathogens from the soil, water, and biomass to food and humans; (iii) the spread of antibiotic resistance genes; and (iv) the limited evaluation methodologies and frameworks with an interdisciplinary approach encompassing the environment, animals, and human health. More research with a significant policy interface is needed to develop targets and roadmaps in national, regional, and global visions [68]. Research should be articulated with economic, social, and environmental stakeholders to improve the scope of current impact monitoring and assessment frameworks and methods, such as environmental strategy, environmental impact, social impact, and life cycle assessments [53,69].

Finally, an innovatively tangible future in circular horticulture requires government actions with financial investments that leverage research. Target 12.3 of the Sustainable Development Goals seeks to halve global food waste at retail and consumer levels and reduce food loss during production and supply [70]. There the Food Waste Index is suggested as a methodology to monitor progress. Although results driven by this target have been reported, several studies have recommended specifying critical issues by sector, redefining the index, and increasing its scope [71,72], and there is a need to prioritize the consolidation of circular food systems more broadly in global environmental initiatives.

We acknowledge two limitations of our research approach that are intrinsic to the nature of bibliometrics. The first limitation is associated with the language. The present study was conducted solely considering publications written in English, and therefore there is an underrepresentation of non-English speaking publications. The second limitation is the sole use of peer-reviewed publication databases, which excludes gray literature and non-indexed journals. Nonetheless, these two sources were used for the definition of the search equation and the discussion of the results.

## 5. Conclusions

Circular horticulture is a recent research field that is gaining attention. Europe has led the dissemination of knowledge. The most productive countries, institutions, and authors are Spain, Italy, and Belgium; the University of Almería; and Bart Vandecashteele and Jane Debode. The most outstanding research topics of circular horticulture are bioeconomy, urban agriculture, nutrient recovery, soilless farming, biochar, fertigation, and desalination.

Current research has prioritized fruits and vegetables as study crops. The circulation strategies have been aligned with biowaste recovery and water reuse. Multiple biowastes from animals and crops have been investigated, which provide benefits as biofertilizers and substitutes for linear substrates. Water management has featured innovations in wastewater treatments, hydroponic systems, and automated technologies. Understanding the One Health approach shows substantial gaps, in which methodologies and evaluation frameworks with an interdisciplinary approach that cover the health of ecosystems, animals, and people are lacking. Greenhouse technologies have shown progress in the development and incorporation of sustainable materials, as well as bioenergy sources, for microclimatic management.

Some challenges that need to be addressed to innovate future research directions in circular horticulture lie in promoting a systemic or integrative perspective to redesign horticultural production systems circularly; formulating circularity indicators; investigating aspects of the collection, transport, and storage of biowaste; training all actors in horticultural value chains on the positive impact of the proper reuse of biowaste; broaden the scope of impact assessment methods framed in the life cycle; and prioritize the consolidation of One Health and circular food systems integrated initiatives.

## Data Availability

The data presented in this paper are available on request from the corresponding author.

## Supplementary Materials

The following supporting information can be downloaded at: https://www.mdpi.com/article/10.3390/ijerph191912053/s1, Table S1. Publications on circular horticulture that were analyzed with the evaluation framework of bibliometric and text mining. Table S2. Text mining analysis search strategy. Table S3. Top 10 most cited publications on circular horticulture.

## References

[1] Hamam M., Chinnici G., Di Vita G., Pappalardo G., Pecorino B., Maesano G., D’Amico M. (2021). Circular economy models in agro-food systems: A review. Sustainability.

[2] UNEP (United Nations Environment Programme) (2021). The Food Waste Index Report 2021.

[3] Bodirsky B.L., Chen D.M.C., Weindl I., Soergel B., Beier F., Molina E.J., Gaupp F., Popp A., Lotze-Campen H. (2022). Integrating degrowth and efficiency perspectives enables an emission-neutral food system by 2100. Nat. Food.

[4] Aznar-Sánchez J.A., Mendoza J.M.F., Ingrao C., Failla S., Bezama A., Nemecek T., Gallego-Schmid A. (2020). Indicators for circular economy in the agri-food sector. Resour. Conserv. Recycl..

[5] Velasco-Muñoz J.F., Mendoza J.M.F., Aznar-Sánchez J.A., Gallego-Schmid A. (2021). Circular economy implementation in the agricultural sector: Definition, strategies and indicators. Resour. Conserv. Recycl..

[6] Geissdoerfer M., Savaget P., Bocken N.M., Hultink E.J. (2017). The Circular Economy—A new sustainability paradigm?. J. Clean. Prod..

[7] Kirchherr J., Reike D., Hekkert M. (2017). Conceptualizing the circular economy: An analysis of 114 definitions. Resour. Conserv. Recycl..

[8] Tseng M.L., Chiu A.S., Chien C.F., Tan R.R. (2019). Pathways and barriers to circularity in food systems. Resour. Conserv. Recycl..

[9] Grumbine R.E., Xu J., Ma L. (2021). An overview of the problems and prospects for circular agriculture in sustainable food systems in the Anthropocene. Circ. Agric. Syst..

[10] Batlles-delaFuente A., Abad-Segura E., González-Zamar M.D., Cortés-García F.J. (2022). An Evolutionary Approach on the Framework of Circular Economy Applied to Agriculture. Agronomy.

[11] Morin A., Katsoulas N., Desimpelaere K., Kartalainen S., Schneegans A. (2019). Circular Horticulture-Starting Paper. EIP-AGRI. http://www.eip-agri_fg_circular_horticulture_starting_paper_2017_en.pdf.

[12] Atzori G., Pane C., Zaccardelli M., Cacini S., Massa D. (2021). The Role of Peat-Free Organic Substrates in the Sustainable Management of Soilless Cultivations. Agronomy.

[13] Carricondo-Martínez I., Berti F., Salas-Sanjuán M.D.C. (2022). Different Organic Fertilisation Systems Modify Tomato Quality: An Opportunity for Circular Fertilisation in Intensive Horticulture. Agronomy.

[14] Massa D., Magán J., Montesano F., Tzortzakis N. (2020). Minimizing water and nutrient losses from soilless cropping in southern Europe. Agric. Water Manag..

[15] World Health Organization (2018). Circular Economy and Health: Opportunities and Risks.

[16] NIHR Global Health Research Unit on Genomic Surveillance of AMR (2020). Whole genome sequencing as part of national and international surveillance programmes for antimicrobial resistance: A roadmap. BMJ Glob. Health.

[17] Castellanos L.R., Van Der Graaf-Van Bloois L., Donado-Godoy P., León M., Clavijo V., Arévalo A., Hordijk J. (2018). Genomic Characterization of Extended-Spectrum Cephalosporin-Resistant Salmonella Enterica in the Colombian Poultry Chain. Front. Microbiol..

[18] Sun Y., Snow D., Walia H., Li X. (2021). Transmission Routes of the Microbiome and Resistome from Manure to Soil and Lettuce. Environ. Sci. Technol..

[19] Yan Z., Xiong C., Liu H., Singh B.K. (2022). Sustainable Agricultural Practices Contribute Significantly to One Health. J. Sustain. Agric. Environ..

[20] WHO Regional Office for Europe (2016). Waste and Human Health: Evidence and Needs: Meeting Report.

[21] Mackenzie J.S., Jeggo M. (2019). The One Health approach—Why is it so important?. Trop. Med. Infect. Dis..

[22] Food and Agricultural Organization of the United Nations Rome Italy (2020). Climate and Sustainable Food Systems. https://www.fao.org/documents/card/en/c/ca6767en/.

[23] Abramo G., D’Angelo C.A., Reale E. (2019). Peer review versus bibliometrics: Which method better predicts the scholarly impact of publications?. Scientometrics.

[24] Donthu N., Kumar S., Mukherjee D., Pandey N., Lim W.M. (2021). How to conduct a bibliometric analysis: An overview and guidelines. J. Bus. Res..

[25] Salloum S.A., Al-Emran M., Monem A.A., Shaalan K. (2018). Using Text Mining Techniques for Extracting Information from Research Articles. Intelligent Natural Language Processing: Trends and Applications.

[26] Mongeon P., Paul-Hus A. (2016). The journal coverage of Web of Science and Scopus: A comparative analysis. Scientometrics.

[27] Allison P.D., de Solla Price D., Griffith B.C., Moravcsik M.J., Stewart J.A. (1976). Lotka’s Law: A Problem in Its Interpretation and Application. Soc. Stud. Sci.

[28] Dayeen F.R., Sharma A.S., Derrible S.A. (2020). Text mining analysis of the climate change literature in industrial ecology. J. Ind. Ecol..

[29] Herrero M., Thornton P.K., Mason-D’Croz. D., Palmer. J., Benton. T.G., Bodirsky. B.L., West P.C. (2020). Innovation can accelerate the transition towards a sustainable food system. Nat. Food.

[30] Yang G., Li J., Liu Z., Zhang Y., Xu X., Zhang H., Xu Y. (2022). Research Trends in Crop-Livestock Systems: A Bibliometric Review. Int. J. Environ. Res. Public Health.

[31] Hartley K., van Santen R., Kirchherr J. (2020). Policies for transitioning towards a circular economy: Expectations from the European Union (EU). Resour. Conserv. Recycl..

[32] Clark M., Springmann M., Rayner M., Scarborough P., Hill J., Tilman D., Harrington R.A. (2022). Estimating the environmental impacts of 57,000 food products. Proc. Natl. Acad. Sci. USA.

[33] Bigdeloo M., Teymourian T., Kowsari E., Ramakrishna S., Ehsani A. (2021). Sustainability and circular economy of food wastes: Waste reduction strategies, higher recycling methods, and improved valorization. Mater. Circ. Econ..

[34] Ahuja I., Dauksas E., Remme J.F., Richardsen R., Løes A.K. (2020). Fish and fish waste-based fertilizers in organic farming—With status in Norway: A review. Waste Manag..

[35] De Corato U. (2020). Agricultural waste recycling in horticultural intensive farming systems by on-farm composting and compost-based tea application improves soil quality and plant health: A review under the perspective of a circular economy. Sci. Total Environ..

[36] Lanauskas J., Uselis N., Buskiene L., Mazeika R., Staugaitis G., Kviklys D. (2021). Cattle horn shavings: A possible nitrogen source for apple trees. Agronomy.

[37] Romero-Perdomo F., Ocampo-Gallego J., Camelo-Rusinque M., Bonilla R. (2019). Lant Growth Promoting Rhizobacteria and Their Potential as Bioinoculants on *Pennisetum clandestinum* (Poaceae). Rev. Biol. Trop..

[38] Mendoza-Labrador J., Romero-Perdomo F., Abril J., Hernández J.P., Uribe-Vélez D., Buitrago R.B. (2021). *Bacillus strains* immobilized in alginate macrobeads enhance drought stress adaptation of guinea grass. Rhizosphere.

[39] Chorolque A., Pellejero G., Sosa M.C., Palacios J., Aschkar G., García-Delgado C., Jiménez-Ballesta R. (2022). Iological Control of Soil-Borne Phytopathogenic Fungi through Onion Waste Composting: Implications for Circular Economy Perspective. Int. J. Environ. Sci. Technol..

[40] Fritsch C., Staebler A., Happel A., Cubero Márquez M.A., Aguiló-Aguayo I., Abadias M., Gallur M., Cigognini I.M., Montanari A., López M.J. (2017). Processing, Valorization and Application of Bio-Waste Derived Compounds from Potato, Tomato, Olive and Cereals: A Review. Sustainability.

[41] Dorr E., Goldstein B., Horvath A., Aubry C., Gabrielle B. (2021). Environmental impacts and resource use of urban agriculture: A systematic review and meta-analysis. Environ. Res. Lett..

[42] Amery F., Debode J., Ommeslag S., Visser R., De Tender C., Vandecasteele B. (2021). Biochar for circular horticulture: Feedstock related effects in soilless cultivation. Agronomy.

[43] Chemetova C., Mota D., Fabião A., Gominho J., Ribeiro H. (2021). Low-Temperature Hydrothermally Treated *Eucalyptus globulus* Bark: From by-Product to Horticultural Fiber-Based Growing Media Viability. J. Clean. Prod..

[44] Vandecasteele B., Muylle H., De Windt I., Van Acker J., Ameloot N., Moreaux K., Debode J. (2018). Plant Fibers for Renewable Growing Media: Potential of Defibration, Acidification or Inoculation with Biocontrol Fungi to Reduce the N Drawdown and Plant Pathogens. J. Clean. Prod..

[45] Chew K.W., Chia S.R., Yen H.W., Nomanbhay S., Ho Y.C., Show P.L. (2019). Transformation of biomass waste into sustainable organic fertilizers. Sustainability.

[46] García-Caparros P., Contreras J.I., Baeza R., Segura M.L., Lao M.T. (2017). Integral management of irrigation water in intensive horticultural systems of Almería. Sustainability.

[47] Ragaveena S., Shirly Edward A., Surendran U. (2021). Smart Controlled Environment Agriculture Methods: A Holistic Review. Rev. Environ. Sci. Bio/Technol..

[48] Lahlou F., Mackey H., Al-Ansari T. (2022). Role of Wastewater in Achieving Carbon and Water Neutral Agricultural Production. J. Clean. Prod..

[49] Jama-Rodzenska A., Sowinski J., Koziel J., Bialowiec A. (2021). Phosphorus recovery from sewage sludge ash based on cradle-cradle approach—Mini-review. Minerals.

[50] Wang F., Fu Y.H., Sheng H.J., Topp E., Jiang X., Zhu Y.G., Tiedje J.M. (2021). Antibiotic Resistance in the Soil Ecosystem: A One Health Perspective. Curr. Opin. Environ. Sci. Health.

[51] McEwen S.A., Collignon P.J. (2018). Antimicrobial Resistance: A One Health Perspective. Microbiol. Spectr..

[52] Ebenso B., Out A., Giusti A., Cousin P., Adetimirin V., Razafindralambo H., Effa E., Gkisakis V., Thiare O., Levavasseur V. (2022). Nature-Based One Health Approaches to Urban Agriculture Can Deliver Food and Nutrition Security. Front. Nutr..

[53] Paris J.M.G., Falkenberg T., Nöthlings U., Heinzel C., Borgemeister C., Escobar N. (2022). Changing dietary patterns is necessary to improve the sustainability of Western diets from a One Health perspective. Sci. Total Environ..

[54] Scherer L., Tomasik B., Rueda O., Pfister S. (2018). Framework for integrating animal welfare into life cycle sustainability assessment. Int. J. Life Cycle Assess..

[55] Romero-Perdomo F., Carvajalino-Umaña J.D., Moreno-Gallego J.L., Ardila N., González-Curbelo M.Á. (2022). Research Trends on Climate Change and Circular Economy from a Knowledge Mapping Perspective. Sustainability.

[56] Villagran E., Bojacá C., Akrami M. (2021). Contribution to the sustainability of agricultural production in greenhouses built on slope soils: A numerical study of the microclimatic behavior of a typical Colombian structure. Sustainability.

[57] Jain A., Sarsaiya S., Awasthi M.K., Singh R., Rajput R., Mishra U.C., Shi J. (2022). Bioenergy and bio-products from bio-waste and its associated modern circular economy: Current research trends, challenges, and future outlooks. Fuel.

[58] De Pascale S., Rouphael Y., Cirillo V., Esposito M., Maggio A. (2021). Modular Systems to Foster Circular Economy in Agriculture. ISHS Acta Horticulturae 1320: VIII South-Eastern Europe Symposium on Vegetables and Potatoes.

[59] Sayadi-Gmada S., Rodríguez-Pleguezuelo C.R., Rojas-Serrano F., Parra-López C., Parra-Gómez S., García-García M.D.C., Manrique-Gordillo T. (2019). Inorganic waste management in greenhouse agriculture in Almeria (SE Spain): Towards a circular system in intensive horticultural production. Sustainability.

[60] Achour Y., Ouammi A., Zejli D. (2021). Technological progresses in modern sustainable greenhouses cultivation as the path towards precision agriculture. Renew. Sustain. Energy Rev..

[61] Nagatoshi Y., Fujita Y. (2019). Accelerating soybean breeding in a CO_2_-supplemented growth chamber. Plant Cell Physiol..

[62] Radenahmad N., Azad A.T., Saghir M., Taweekun J., Bakar M.S.A., Reza M.S., Azad A.K. (2020). A review on biomass derived syngas for SOFC based combined heat and power application. Renew. Sustain. Energy Rev..

[63] Rigamonti L., Mancini E. (2021). Life cycle assessment and circularity indicators. Int. J. Life Cycle Assess..

[64] Dayioğlu M., Turker U. (2021). Digital transformation for sustainable future—Agriculture 4.0: A review. J. Agric. Sci..

[65] Fangueiro D., Alvarenga P., Fragoso R. (2021). Horticulture and orchards as new markets for manure valorisation with less environmental impacts. Sustainability.

[66] Platform for Accelerating the Circular Economy—PACE. https://pacecircular.org/action-agenda/food.

[67] Willett W., Rockström J., Loken B., Springmann M., Lang T., Vermeulen S., Murray C.J. (2019). Food in the Anthropocene: The EAT–Lancet Commission on healthy diets from sustainable food systems. Lancet.

[68] Ikhimiukor O.O., Odih E.E., Donado-Godoy P., Okeke I.N. (2022). A Bottom-up View of Antimicrobial Resistance Transmission in Developing Countries. Nat. Microbiol..

[69] World Health Organization (2019). Assessing the Health Impacts of a Circular Economy (No. WHO/EURO: 2019-3504-43263-60634).

[70] Gasper D., Shah A., Tankha S. (2019). The framing of sustainable consumption and production in SDG 12. Glob. Policy.

[71] Okayama T., Watanabe K., Yamakawa H. (2021). Sorting Analysis of Household Food Waste—Development of a Methodology Compatible with the Aims of SDG12. Sustainability.

[72] Bryan B.A., Hadjikakou M., Moallemi E.A. (2019). Rapid SDG progress possible. Nat. Sustain..

