# Supplementary material for: Insights into Circular Horticulture: Knowledge Diffusion, Resource Circulation, One Health Approach, and Greenhouse Technologies
Source: Int J Environ Res Public Health. 2022 Sep 23;19(19):12053. doi: 10.3390/ijerph191912053 (PMC9565935; doi:10.3390/ijerph191912053)
Supplement: Supplementary file 1 [file ijerph-19-12053-s001.zip › ijerph-1878711-supplementary.pdf]

International Journal of
Environmental Research and Public Health

MDPI

Insights into Circular Horticulture: Knowledge Diffusion, Resource Circulation, One Health Approach, and Greenhouse Technologies

Diego Alejandro Salinas-Velandia, Felipe Romero-Perdomo, Stephanie Numa-Vergel, Edwin Villagrán, Pilar Donado-Godoy and Julio Ricardo Galindo-Pacheco

Supplementary Materials

Table S1. Publications on circular horticulture that were analyzed with the evaluation framework of bibliometric and text mining.

Title	Authors	Year	Journal	Country
Exploring the agricultural reutilisation of desalination reject brine from reverse osmosis technology	Jiménez-Arias, D., Sierra, S. M., García-Machado, F. J., García-García, A. L., Borges, A. A., & Luis, J. C.	2022	Desalination	Spain
Biochar for intensification of plant-related industries to meet productivity, sustainability and economic goals: A review	Kochanek, J., Soo, R. M., Martinez, C., Dakuidreketi, A., & Mudge, A. M.	2022	Resources, Conservation and Recycling	Australia
How decentralized treatment can contribute to the symbiosis between environmental protection and resource recovery	Estévez, S., González-García, S., Feijoo, G., & Moreira, M. T.	2022	Science of the Total Environment	Spain
Biodisinfection as a profitable fertilization method for horticultural crops in the framework of the circular economy	Castillo-Díaz, F. J., Belmonte-Ureña, L. J., Camacho-Ferre, F., & Tello Marquina, J. C.	2022	Agronomy	Spain
Peaches detection using a deep learning technique—A contribution to yield estimation, resources management, and circular economy	Assunção, E. T., Gaspar, P. D., Mesquita, R. J., Simões, M. P., Ramos, A., Proença, H., & Inacio, P. R.	2022	Climate	Portugal
Biorefining within food loss and waste frameworks: a review	Jones, R. E., Speight, R. E., Blinco, J. L., & O'Hara, I. M.	2022	Renewable and Sustainable Energy Reviews	Australia
Review of the potential for recycling CO_2 from organic waste composting into plant production under controlled environment agriculture	Thomson, A., Price, G. W., Arnold, P., Dixon, M., & Graham, T.	2021	Journal of Cleaner Production	Canada
The effect of phosgreen fertilization on the growth and phosphorus uptake of lettuce (*Lactuca sativa*)	Jama-Rodzeńska, A.	2022	International Journal of Agriculture & Biology	Poland

Biofertilizers and biostimulants for agricultural and aquaculture use: bioprocesses applied to organic by-products of the fishing industry [biofertilizantes y bioestimulantes para uso agrícola y acuícola: bioprocesos aplicados a subproductos orgánicos de la industria pesquera]	Florez-Jalixto, M., Roldán-Acero, D., Omote-Sibina, J. R., & Molleda-Ordoñez, A.	2021	Scientia Agropecuaria	Peru
Bioactive modified non-wovens as a novel approach of plants protection against invasive slugs	Rosowski, M., Puchowicz, D., Jaskulska, M., Kozłowski, J., & Cieślak, M.	2021	Materials	Poland
Use of recovered struvite and ammonium nitrate in fertigation in tomato (*Lycopersicum esculentum*) production for boosting circular and sustainable horticulture	Carreras-Sempere, M., Caceres, R., Viñas, M., & Biel, C.	2021	Agriculture	Spain
Evaluating recycling fertilizers for tomato cultivation in hydroponics, and their impact on greenhouse gas emissions	Halbert-Howard, A., Häfner, F., Karlowsky, S., Schwarz, D., & Krause, A.	2021	Environmental Science and Pollution Research	Germany
Low-temperature hydrothermally treated eucalyptus globulus bark: from by-product to horticultural fiber-based growing media viability	Chemetova, C., Mota, D., Fabião, A., Gominho, J., & Ribeiro, H.	2021	Journal of Cleaner Production	Netherlands, Portugal
Significance and vision of nutrient recovery for sustainable city food systems in Germany by 2050	Jiménez-Arias, D., Sierra, S. M., García-Machado, F. J., García-García, A. L., Borges, A. A., & Luis, J. C.	2022	Desalination	Spain
Reuse of the spent mushroom substrate in a vegetable seedbed [reutilización del sustrato post-cultivo de hongos en semillero de hortícolas]	Yague-Carrasco, M. R., & Carmen Lobo, M.	2021	ITEA—Informacion Tecnica Economica Agraria	Spain
Phosphorus recovery from sewage sludge ash based on cradle-to-cradle approach—mini-review	Jama-Rodzeńska, A., Sowiński, J., Koziel, J. A., & Białowiec, A.	2021	Minerals	Poland, USA
Gasification biochar from horticultural waste: an exemplar of the circular economy in Singapore	Arora, S., Jung, J., Liu, M., Li, X., Goel, A., Chen, J., ... & Wang, C. H.	2021	Science of The Total Environment	Singapore, China, Australia
Innovative strategy to reduce single-use plastics in sustainable horticulture by a refund strategy for flowerpots	Blanke, M. M., & Golombek, S. D.	2021	Sustainability	Germany
The role of peat-free organic substrates in the sustainable management of soilless cultivations	Atzori, G., Pane, C., Zaccardelli, M., Cacini, S., & Massa, D.	2021	Agronomy	Italy
Chemically versus thermally processed brown shrimp shells or chinese mitten crab as a source of chitin, nutrients or salts and as microbial stimulant in soilless strawberry cultivation	Vandecasteele, B., Amery, F., Ommeslag, S., Vanhoutte, K., Visser, R., Robbens, J., ... & Debode, J.	2021	Science of the Total Environment	Belgium, The Netherlands

The evolution of soilless systems towards ecological sustainability in the perspective of a circular economy. Is it really the opposite of organic agriculture?	Gonnella, M., & Renna, M.	2021	Agronomy	Italy
Biochar for circular horticulture: feedstock related effects in soilless cultivation	Amery, F., Debode, J., Ommeslag, S., Visser, R., De Tender, C., & Vandecasteele, B.	2021	Agronomy	Belgium, The Netherlands,
The role of biotechnology in the transition from plastics to bioplastics: an opportunity to reconnect global growth with sustainability	Degli Esposti, M., Morselli, D., Fava, F., Bertin, L., Cavani, F., Viaggi, D., & Fabbri, P.	2021	FEBS Open Bio	Italy
Bioeconomy as a transforming driver of intensive greenhouse horticulture in Spain	Egea, F. J., López-Rodríguez, M. D., Oña-Burgos, P., Castro, A. J., & Glass, C. R.	2021	New Biotechnology	Spain, USA, United Kingdom
Cattle horn shavings: a possible nitrogen source for apple trees	Lanauskas, J., Uselis, N., Buskienė, L., Mažeika, R., Staugaitis, G., & Kviklys, D.	2021	Agronomy	Lithuania, Norway
Biochar industry to circular economy	Hu, Q., Jung, J., Chen, D., Leong, K., Song, S., Li, F., ... & Wang, C. H.	2021	Science of the Total Environment	Singapore, Australia, South Korea, China
Increasing city resilience through urban agriculture: challenges and solutions in the global north	Gulyas, B. Z., & Edmondson, J. L.	2021	Sustainability	United Kingdom
Horticulture and orchards as new markets for manure valorisation with less environmental impacts	Fangueiro, D., Alvarenga, P., & Fragoso, R.	2021	Sustainability	Portugal
Aqueous extracts and residual biomass use in sustainable agriculture: a circular economy model	Beni, C., Rinaldi, S., Masciarelli, E., Di Luigi, M., Casorri, L., & Neri, U.	2021	Environmental Engineering and Management Journal	Italy
Recent advances in extraction technologies for recovery of bioactive compounds derived from fruit and vegetable waste peels: a review	Rifna, E. J., Misra, N. N., & Dwivedi, M.	2021	Critical Reviews in Food Science and Nutrition	India, Canada
Calcium phosphate particles coated with humic substances: a potential plant biostimulant from circular economy	Adamiano, A., Fellet, G., Vuerich, M., Scarpin, D., Carella, F., Piccirillo, C., ... & Iafisco, M.	2021	Molecules	Italy
Strawberry fields forever: that is, how many grams of plastics are used to grow a strawberry?	Galati, A., Sabatino, L., Prinzivalli, C. S., D'Anna, F., & Scalenghe, R.	2020	Journal of Environmental Management	Italy
Grow–store–steam–repeat: reuse of spent growing media for circular cultivation of chrysanthemum	Vandecasteele, B., Blindeman, L., Amery, F., Pieters, C.,	2020	Journal of Cleaner Production	Belgium

	Ommeslag, S., Van Loo, K., ... & Debode, J.			
What horticulture and space exploration can learn from each other: the mission to mars initiative in The Netherlands	Vermeulen, A. C., Hubers, C., de Vries, L., & Brazier, F.	2020	Acta Astronautica	The Netherlands
Minimizing water and nutrient losses from soilless cropping in Southern Europe	Massa, D., Magán, J. J., Montesano, F. F., & Tzortzakis, N.	2020	Agricultural water management	Italy, Spain, Cyprus
Agricultural waste recycling in horticultural intensive farming systems by on-farm composting and compost-based tea application improves soil quality and plant health: a review under the perspective of a circular economy	De Corato, U.	2020	Science of the Total Environment	Italy
Identification of opportunities for applying the circular economy to intensive agriculture in Almería (South-East Spain)	Aznar-Sánchez, J. A., Velasco-Muñoz, J. F., García-Arca, D., & López-Felices, B.	2020	Agronomy	Spain
Boosting leaf contents of rutin and solanesol in bio-waste of *Solanum lycopersicum*	Röhlen-Schmittgen, S., Ellenberger, J., Groher, T., & Hunsche, M.	2020	Plant Physiology and Biochemistry	Germany, Switzerland
Biodegradable raffia as a sustainable and cost-effective alternative to improve the management of agricultural waste biomass	Duque-Acevedo, M., Belmonte-Ureña, L. J., Toresano-Sánchez, F., & Camacho-Ferre, F.	2020	Agronomy	Spain
Optimization of high-pressure extraction process of antioxidant compounds from feteasca regala leaves using response surface methodology	Becze, A., Babalau-Fuss, V. L., Varaticeanu, C., & Roman, C.	2020	Molecules	Romania
Fish and fish waste-based fertilizers in organic farming—with status in norway: a review	Ahuja, I., Dauksas, E., Remme, J. F., Richardsen, R., & Løes, A. K.	2020	Waste Management	Norway
Public policies for sustainability and water security: the case of Almeria (Spain)	Caparrós-Martínez, J. L., Rueda-Lópe, N., Milán-García, J., & de Pablo Valenciano, J.	2020	Global Ecology and Conservation	Spain
Storage, fertilization and cost properties highlight the potential of dried microbial biomass as organic fertilizer	Spanoghe, J., Grunert, O., Wambacq, E., Sakarika, M., Papini, G., Alloul, A., ... & Vlaeminck, S. E.	2020	Microbial biotechnology	Belgium
Management of poultry manure in Poland—current state and future perspectives	Dróżdż, D., Wystalska, K., Malińska, K., Grosser, A., Grobelak, A., & Kacprzak, M.	2020	Journal of Environmental Management	Poland
A new network for the advancement of marine biotechnology in Europe and Beyond	Rotter, A., Bacu, A., Barbier, M., Bertoni, F., Bones, A. M., Cancela, M. L., ... & Vasquez, M. I.	2020	Frontiers in marine science	Slovenia, Albania, France, Switzerland, Norway, Portugal, Ireland,

				Poland, Turkey, Greece, Montenegro, Latvia, Iceland, Israel, Italy, Croatia, Lithuania, Estonia, Malta, Denmark, United Kingdom, Germany, Spain, Belgium, Slovenia, Czechia, Estonia, Cyprus
Upcycling phosphorus recovered from anaerobically digested dairy manure to support production of vegetables and flowers	Porterfield, K. K., Joblin, R., Neher, D. A., Curtis, M., Dvorak, S., Rizzo, D. M., ... & Roy, E. D.	2020	Sustainability	USA
Production of spinach in intensive mediterranean horticultural systems can be sustained by organic-based fertilizers without yield penalties and with low environmental impacts	Vico, A., Sáez, J. A., Pérez-Murcia, M. D., Martinez-Tomé, J., Andreu-Rodríguez, J., Agulló, E., ... & Moral, R.	2020	Agricultural Systems	Spain
The management of agricultural waste biomass in the framework of circular economy and bioeconomy: an opportunity for greenhouse agriculture in Southeast Spain	Duque-Acevedo, M., Belmonte-Ureña, L. J., Plaza-Úbeda, J. A., & Camacho-Ferre, F.	2020	Agronomy	Spain
Cotton and cardoon byproducts as potential growing media components for *Cichorium spinosum* L. commercial cultivation	Petropoulos, S., Fernandes, Â., Stojković, D., Pereira, C., Taofiq, O., Di Gioia, F., ... & Ferreira, I. C.	2019	Journal of Cleaner Production	Greece, Portugal, Serbia, USA, Cyprus
Exploring the environmental performance of urban symbiosis for vertical hydroponic farming	Martin, M., Poulikidou, S., & Molin, E.	2019	Sustainability	Sweden
A horticultural medium established from the rapid removal of phytotoxins fromwinery grape marc	Olejar, K. J., Vandermeer, C., Fedrizzi, B., & Kilmartin, P. A.	2019	Horticulturae	New Zealand

Market-based tools for a plastic waste reduction policy in agriculture: a case study in the south of Italy	De Lucia, C., & Pazienza, P.	2019	Journal of Environmental Management	Italy
Six collective challenges for sustainability of Almería greenhouse horticulture	Castro, A. J., López-Rodríguez, M. D., Giagnocavo, C., Gimenez, M., Céspedes, L., La Calle, A., ... & Valera, D. L.	2019	International journal of environmental research and public health	Spain, USA
Influence of supplementary led lighting on physiological and biochemical parameters of tomato (*Solanum lycopersicum* L.) leaves	Groher, T., Röhlen-Schmittgen, S., Fiebig, A., Noga, G., & Hunsche, M.	2019	Scientia Horticulturae	Germany
Circular agri-food approaches: will consumers buy novel products made from vegetable waste?	McCarthy, B., Kapetanaki, A. B., & Wang, P.	2019	Rural Society	Australia, UK, Singapore
Inorganic waste management in greenhouse agriculture in Almeria (Spain): towards a circular system in intensive horticultural production	Sayadi-Gmada, S., Rodríguez-Pleguezuelo, C. R., Rojas-Serrano, F., Parra-López, C., Parra-Gómez, S., García-García, M. D. C., ... & Manrique-Gordillo, T.	2019	Sustainability	Spain
Plant fibers for renewable growing media: potential of defibration, acidification or inoculation with biocontrol fungi to reduce the drawdown and plant pathogens	Vandecasteele, B., Muylle, H., De Windt, I., Van Acker, J., Ameloot, N., Moreaux, K., ... & Debode, J.	2018	Journal of Cleaner Production	Belgium
Microbiota from 'next-generation green compost' improves suppressiveness of composted municipal-solid-waste to soil-borne plant pathogens	De Corato, U., Salimbeni, R., De Pretis, A., Patruno, L., Avella, N., Lacolla, G., & Cucci, G.	2018	Biological Control	Italy
Recycling of P and K in circular horticulture through compost application in sustainable growing media for fertigated strawberry cultivation	Vandecasteele, B., Debode, J., Willekens, K., & Van Delm, T.	2018	European Journal of Agronomy	Belgium
The energy & raw materials factory: role and potential contribution to the circular economy of The Netherlands	van Leeuwen, K., de Vries, E., Koop, S., & Roest, K.	2018	Environmental management	The Netherlands
An efficient agro-industrial complex in almería (Spain): towards an integrated and sustainable bioeconomy model	Egea, F. J., Torrente, R. G., & Aguilar, A.	2018	New biotechnology	Spain, Belgium
Effectiveness of a full-scale horizontal slow sand filter for controlling phytopathogens in recirculating hydroponics: from microbial isolation to full microbiome assessment	Prenafeta-Boldú, F. X., Trillas, I., Viñas, M., Guivernau, M., Cáceres, R., & Marfà, O.	2018	Science of the Total Environment	Spain
Circular economy and economic viability of aquaponic systems: comparing urban, rural and peri-urban scenarios under dutch conditions	Stadler, M. M., Baganz, D., Vermeulen, T., & Keesman, K. J.	2015	Acta Horticulturae	The Netherlands
Farmer perceptions and use of organic waste products as fertilisers—a survey study of potential benefits and barriers	Case, S. D. C., Oelofse, M., Hou, Y., Oenema, O., & Jensen, L. S.	2017	Agricultural Systems	Denmark, The

	Netherlands

Table S2. Text mining analysis search strategy.

Major Topics	Minor Topics	Search Equations
Crops	Vegetables	(Vegetable* OR Cabbage OR Tomato OR Onion OR Eggplant OR Carrot OR Cauliflower OR Kale OR Cucumber OR Garlic OR Leek OR Lettuce OR Spinach OR Beans OR Cowpeas OR Pumpkin OR "field peas" OR Aubergine OR Chili OR "Diplotaxis tenuifolia" OR "Valerianella locusta")
	Fruits	(Fruit* OR Strawberry OR "passion fruit" OR papaya OR jackfruit OR citrus OR pineapple OR apple OR mango OR avocado OR "apple banana" OR bogoya OR watermelon OR guava OR grape OR melon)
	Aromatic plants	(aromatic OR "green pepper" OR "sweet pepper"OR "hot pepper" OR vanilla OR ginger OR turmeric OR marigold)
	Ornamental plants	(Amaranthus OR Rose OR chrysanthemum OR poinsettia OR gerberas OR pansy)
Resource Circulation Strategies	Biowaste	("animal waste" OR "poultry manure" OR "cattle horn shavings" OR "chinese mitten crab" OR "chicken manure" OR "livestock manure" OR "animal manure" OR "duck feces" OR "sheep dung" OR "pig manure" OR "goat manure" OR "cattle manure" OR "food waste" OR compost OR *compost* OR "residual biomass" OR "organic waste" OR "reused plant debris" OR "fiber material")
	Water	(Water OR wastewater OR "reverse osmosis" OR seawater OR desalination OR fertigation OR rainwater OR "soaker hoses" or "water overuse" OR "water use efficiency")
	One Health	("public health" OR Salmonella OR Campylobacter OR Klebsiella OR "Antimicrobial Resistance" OR "Whole-Genome Sequencing" "Global Health" OR Epidemiology OR "Pathogen Surveillance" OR Pathogen OR "Genomic Surveillance" OR "Escherichia coli" OR Enterococcus OR "One health" OR "food safety" OR Enterobacteriaceae OR "animal welfare" OR "human health" OR "environmental health")
Greenhouse technologies	Sensing technologies	("remote sensing" OR "precision agriculture" OR "machine learning" OR "nanotechnology" OR "controlled enviroment agriculture" OR "microclimate" OR "numerical simulation")
	Sustainable materials	("bioplastics" OR "plastic waste" OR "plastic remains" OR "debris plastic" OR "recycle plastic material" OR "sustainable material" OR biopolymer)

Bioenergy	("anaerobic digestion" OR biomass OR biorefinery OR biogass OR biofuels OR "renewble energy" OR biochar OR pyrolysis OR biomethane OR charcoal OR Bi-orefining OR biorefineries OR "wind power" OR agrivoltaic OR "Solar energy" OR "Solar panel" OR "sustainable energy" OR microalgae OR Hydropower OR "Hydraulic energy")

Table S3. Top 10 most cited publications on circular horticulture.

Rank	Document Title	Number of Citations
1	Farmer perceptions and use of organic waste products as fertilisers—A survey study of potential benefits and barriers	61
2	Agricultural waste recycling in horticultural intensive farming systems by on-farm composting and compost-based tea application improves soil quality and plant health: A review under the perspective of a circular economy	57
3	The Energy & Raw Materials Factory: Role and Potential Contribution to the Circular Economy of the Netherlands	49
4	Management of poultry manure in Poland—Current state and future perspectives	45
5	An efficient agro-industrial complex in Almería (Spain): Towards an integrated and sustainable bioeconomy model	38
6	The Management of Agricultural Waste Biomass in the Framework of Circular Economy and Bioeconomy: An Opportunity for Greenhouse Agriculture in Southeast Spain	30
7	Minimizing water and nutrient losses from soilless cropping in southern Europe	28
8	Biochar Industry to Circular Economy	26
9	Six Collective Challenges for Sustainability of Almería Greenhouse Horticulture	24
10	Plant fibers for renewable growing media: Potential of defibration, acidification or inoculation with biocontrol fungi to reduce the N drawdown and plant pathogens	23
